# Chromium-based metal organic framework for pipette tip micro-solid phase extraction: an effective approach for determination of methyl and propyl parabens in wastewater and shampoo samples

**DOI:** 10.1186/s13065-021-00786-7

**Published:** 2021-11-06

**Authors:** Massoud Kaykhaii, Sayyed Hossein Hashemi, Fariba Andarz, Amin Piri, Ghasem Sargazi, Grzegorz Boczkaj

**Affiliations:** 1 Department of Chemistry, Faculty of Sciences, University of Sistan and Baluchestan, Zahedan, 98136-674 Iran; 2 Department of Marine Chemistry, Faculty of Marine Science, Chabahar Maritime University, Chabahar, Iran; 3 Nanomaterial Technology Department, Non-Communicable Diseases Research Centre, Bam University of Medical Sciences, Bam, Iran; 4 Department of Process Engineering and Chemical Technology, Faculty of Chemistry, Gdansk University of Technology, Gdansk, Poland

**Keywords:** Paraben, Pipette tip micro solid phase extraction, Sample preparation, Metal organic framework, Response surface methodology, Spectrophotometry

## Abstract

**Background:**

A chromium-based metal organic framework was synthesized and employed as an efficient sorbent for pipette tip micro-solid phase extraction and preconcentration of parabens from wastewater and shampoo samples up to sub-ppb level before their spectrophotometric analysis.

**Results:**

Factors affecting preconcentration including volume and type of solvent, amount of sorbent, number of extraction, and volume and pH of samples were optimized employing one-variable-at-a-time and response surface methodology. Obtained analytical characteristics of the method proves its usefulness for analysis of real samples. Linear range of the method for parabens was 1.0–200.0 μg/L. Detection limit of the protocol was 0.24 µg/L for propyl paraben and 0.25 µg/L for methyl paraben. Reproducibility of the protocol defined as % RSD was better than 5.78%. Synthesized adsorbent can be re-used for at least 20 extractions.

**Conclusion:**

The method showed a good detection limit and precision for determination of methyl- and propyl-paraben in wastewater and shampoo samples.

**Supplementary Information:**

The online version contains supplementary material available at 10.1186/s13065-021-00786-7.

## Introduction

Parabens are esters derived of *p*-hydroxybenzoic acid with antimicrobial properties which are in use as preservatives in personal care products, especially shampoos to prevent fungal and microbial infections. It is estimated that up to 90% of cosmetics contain parabens at levels of 0.01–0.3% [1]. The most common parabens that exist in cosmetics are methylparaben (MP), propylparaben (PP), ethylparaben and butylparaben. Paraben mixtures are generally added to cover a general antimicrobial spectrum because some of them have selective activity [2].

Wastewater consists of a vast variety of chemicals emerging pollutants. One of them are parabens which may enter to wastewater from personal care products, especially shampoos, and ultimately can contaminate environment. Parabens can also be released into water ecosystems mainly from personal care production plant discharges. Exposure to these compounds may cause decreased hatching in birds, fish and turtles, feminization of male fish, reptiles, birds and mammals; and changes in the immune system of marine mammals; and changes in the immune system of marine mammals because of their bioaccumulation potential. The con increase breast cancer incidence interferes in male reproductive functions and influence malignant melanoma development that exhibits to be influenced using estrogenic stimulation [3, 4].

Several analytical techniques were used for the analysis of parabens in cosmetics and aquatic samples, including: high performance liquid chromatography ultraviolet detection (HPLC–UV) [1, 5], gas chromatography [6], gas chromatography-mass spectrometry [7] and capillary electrophoresis [8, 9]. Because of low concentration of these substances and complexity of media, a sample enrichment/extraction step is indispensable. Solid phase extraction (SPE) [8], ultrasonic nebulization extraction assisted dispersive liquid–liquid microextraction [10], solid phase microextraction [11], dispersive liquid–liquid microextraction [12], matrix solid phase dispersion [13] and magnetically assisted matrix solid phase dispersion [14] are some advanced techniques which have been introduced for enrichment of parabens from different real samples. However, most of them are time consuming, multi-step, need large volumes of samples and adsorbent or they are not economical.

In the sense, modern SPE versions have introduced to miniaturize and simplify the extraction protocols, including pipette tip based micro-SPE (µSPE), because of its inherent advantages which consist of ease of use, reduced solvent consumption and sample volume and high sample throughput applying either multi-channel hand pipettes or robotic liquid handling system [15–22].

Metal organic frameworks (MOFs) are classified as extremely ordered crystalline metal clusters with high porosity (> 90%) and extremely large surface area (up to 7410 m^2^/g), consist of metal–oxide clusters and organic linkers. The variation of metal oxides and the proper selection of organic linkers allow the pore size, volume, and functionality to be tailored for designable applications. Also, the characteristics of MOFs is mainly based on the nature of the selected inorganic and organic nodes and ligands and their connectivity [23]. Owing to their fascinating structures and abnormal properties, including permanent nanoscale porosity, high surface, uniform structure cavities and good thermostability, they found many applications in chemical analysis, mainly as extracting media [24]. MOFs has found some novel application such as extraction of cyanide from different water samples by porous copper based MOF modified with carbon paste electrode [25].

Response surface methodology (RSM) can be summarized as a compilation of statistical tools and procedure for constructing and obtaining function relationship between a response variable and set of design variable. It is the collection of mathematical and numerical techniques that are enough for modelling and analysis of the problems having numerous variables influencing the absorbance as response, and objective is to investigate the response. The most extensive application of RSM can be found in industrial world, where a number of input variables affect some performance determines, named the response, in ways that are not easy or unfeasible to depict using a rigorous mathematical formulation [26, 27].

The aim of this paper is to present a simple, selective, fast and sensitive method for the extraction and preconcentration of MP and PP from wastewater and shampoo samples using a novel metal organic frameworks pipette tip micro-solid phase extraction (MOF PT-µSPE). A regular spectrophotometer was employed for quantitative analysis. The protocol was optimized utilizing one-variable-at-a-time and RSM.

## Experimental

### Apparatus

Spectrophotometric determinations were performed on a PerkinElmer model Lambda 25 double beam spectrophotometer (US) at a wavelength of 255 nm for both parabens. Microcells with 20 µL capacity were employed. For pH adjustments, a Metrohm (Switzerland) pH meter model 713 was employed. For qualitative spectral interpretation and structural elucidation of MOFs, Fourier transform infrared (FTIR) spectrometer (Bucks, UK) was used. A Bruker (model D8 Advance, Germany) was employed for powder X-ray diffraction (XRD) measurements. The morphology of MOFs was investigated using a scanning electron microscope (SEM), model MIRA3 TESCAN (Czech Republic).

### Reagents

All chemicals were of analytical grade (Merck KGaA, Darmstadt, Germany) with high purity and applied as received. Purity of main reagents such as 2,6-pyridine dicarboxylic acid and Cr(NO_3_)_3_∙4H_2_O were 99%. During all experiments, ultra-pure water (18.2 MΩ), purified by MilliQ-Millipore system (Millipore, Germany) was used. Fresh stock standard solutions of 100 mg/L of the parabens was made daily by dissolving 100 mg of the reagents in 1000 mL of water, that was maintained away from light in a refrigerator. Working standard solutions were achieved by dilution of these stock solutions before analysis.

### Synthesis of Cr-MOF adsorbent

Microwave technique was used to prepare this novel Cr-MOF adsorbent for the first time. At first, 0.02 mol of Cr(NO_3_)_2_ and 0.06 mol of pyridine-2,6 dicarboxylic acid (0.06 mmol) ligand were separately dissolved in 25 mL of water. Ligand solution was slowly added to the chromium nitrate and mixture was stirred approximately 40 min at 85 °C. In the next step, the Cr-MOF sample nanostructures were entered into a microwave reactor and placed under the optimal irradiation with the microwave power of 600 W for 10 min at the ambient temperature. After cooling to room temperature crystals of porous Cr-MOF were formed, which was washed with distilled water and separated by filtration.

### Extraction procedure

In order to prepare a column for pipette tip micro-solid phase extraction, the suitable amount of the MOF was packed loosely in a pipette-tip as discussed in one of our previous papers [28]. Cotton was put at both ends of the tip to avoid adsorbent loss. Before the first use, this column was washed ten times by 10.0 mL portion of ultrapure water using a commercial syringe. For the purpose of optimization of the extraction, a 34.0 mL aliquot of sample (pH = 7.0) was spiked by paraben to make a 180.0 µg/L solution of each analyte and 100 µL of it was loaded to the tip 7 times. Then, the analyte were eluted by 100 µL of chloroform (eluent solvent) for 4 times. Each time, the eluent was recycled to the same vial. Finally, 15 µL of the desorbed analytes were transferred to a quartz micro-cell and determined by a spectrophotometer.

### Preparation of real samples

In order to assess the applicability of suggested protocol to real samples in a complicated matrix, it was applied to extract and enrich MP and PP in wastewater and shampoo samples. Wastewater taken from the sewage of the University of Sistan and Baluchestan. It was filtered through a filter paper before extraction to be free from suspending particles. For shampoo sample, 0.3 g of it was weighed in a flask and dissolved in 100 mL ultra-pure water. Next, 25 mL of the solution was diluted to 100 mL and used for PT-µSPE. No analytes were found in the selected samples (confirmed by HPLC–UV)—which was also in agreement with composition declared by the shampoo manufacturer.

## Results and discussion

### Characterization of the sorbent

**Fig. 1:**
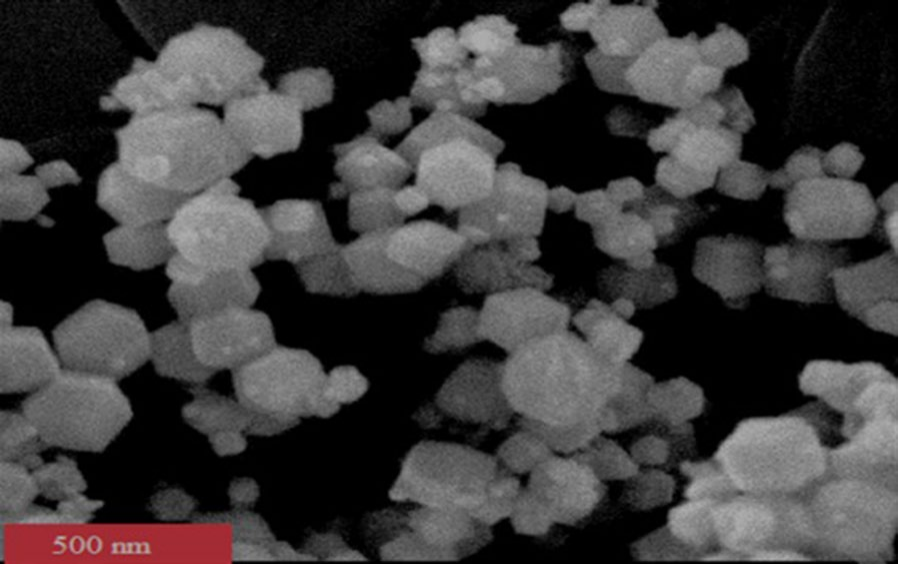
Scanning electron microscope image of the synthesized Cr-MOF sorbent

The SEM image of the synthesized Cr-MOF is depicted in Fig. 1. As can be seen, it has a pure uniform size distribution as well as a homogenous morphology and there is no sign of the agglomeration or aggregation in it. The creation of the pores is visible in the image, and the average size of the synthesized Cr-MOF is 50 nm. Using Barrett–Joyner–Halenda (BJH) technique, the specific surface area of the sorbent was determined as 2.8 × 10^2^ m^2^/g.

**Fig. 2:**
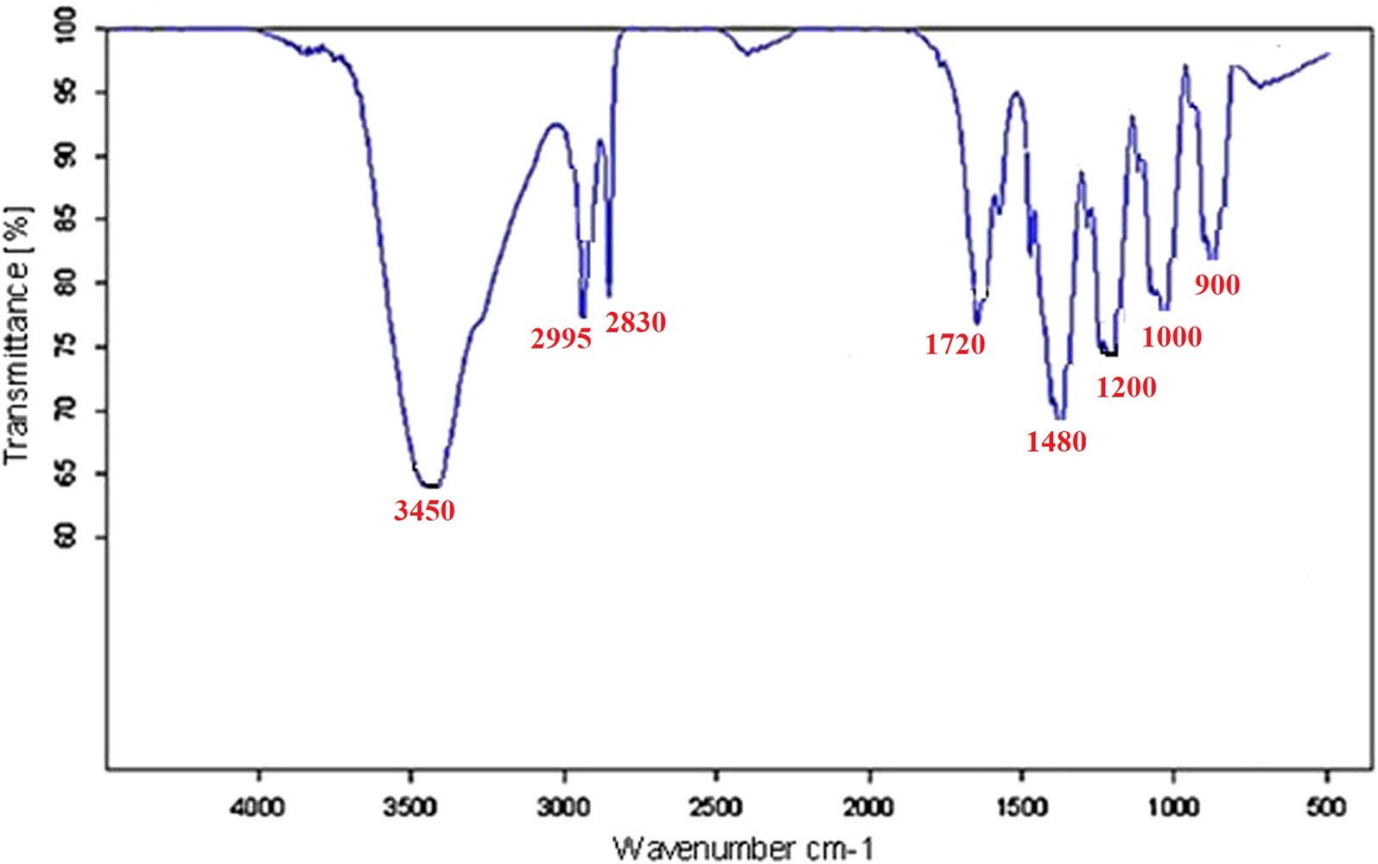
FTIR spectrum of Cr-MOF sorbent

**Fig. 3:**
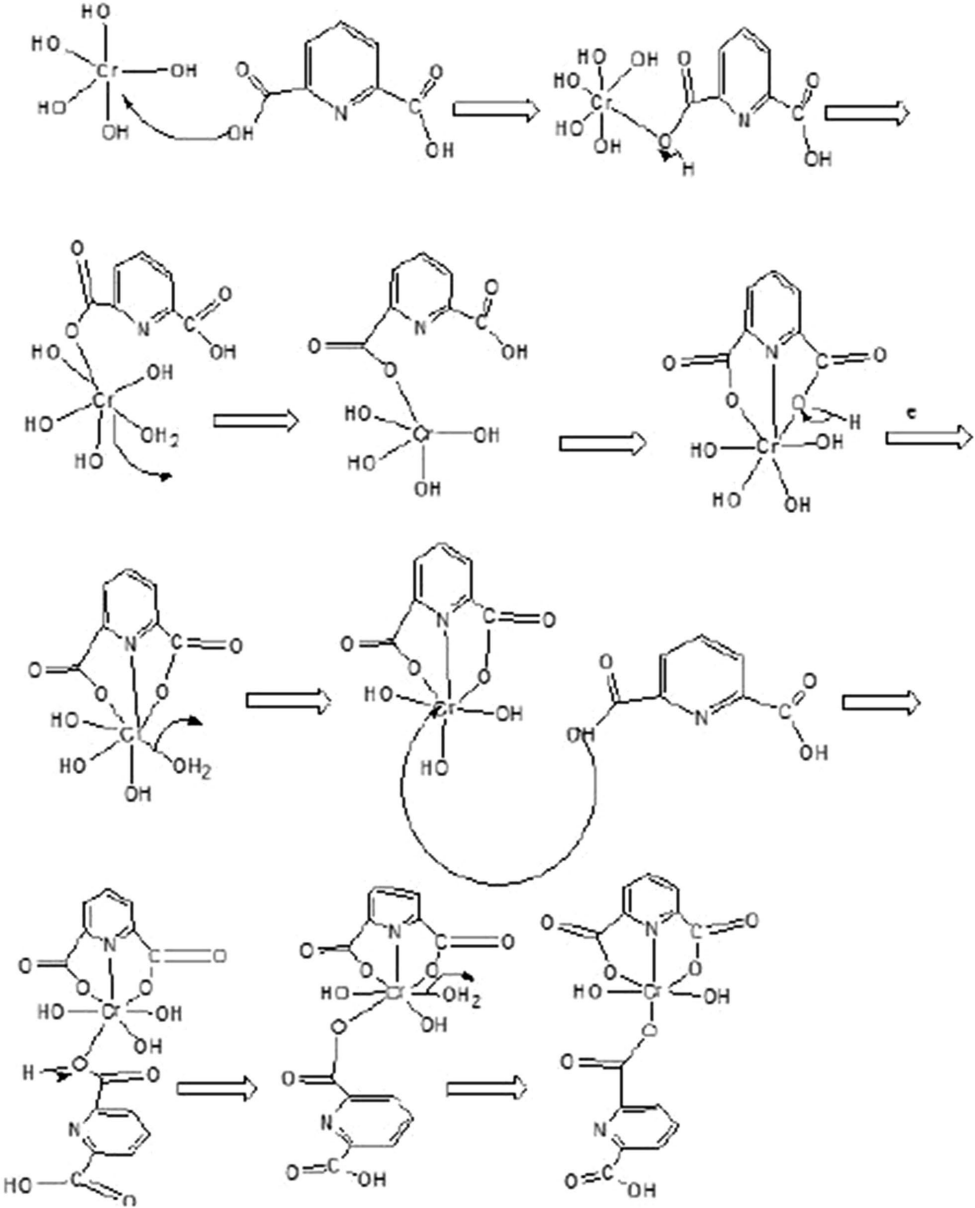
Proposed mechanism for the formation of Cr-MOF sorbent

**Fig. 4:**
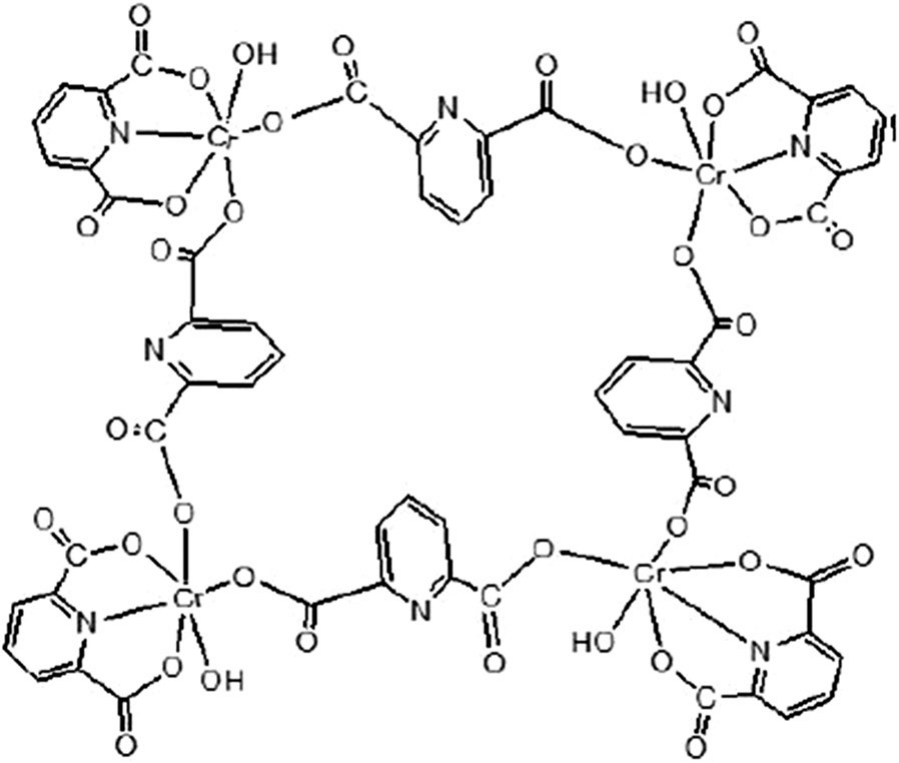
The proposed structure related to formation of the Cr-MOF sorbent

**Fig. 5:**
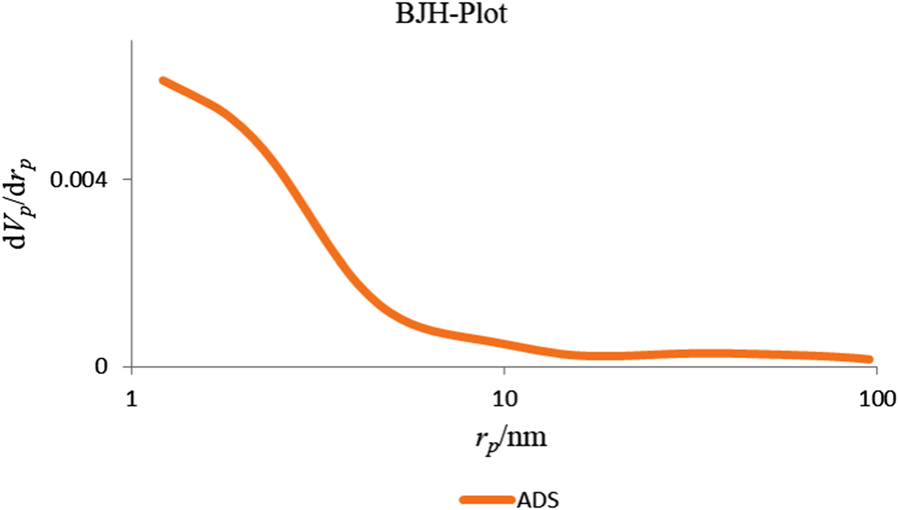
BJH pore size distribution of Cr-MOF nanostructures

The FTIR spectrum of Cr-MOF is shown on Fig. 2. The broad band in the region of 3500 cm^−1^ reveals the presence of coordinated water in the structure. Also, wave number values in the regions of 2830 cm^−1^ and 2995 cm^−1^ confirmed CH_2_ aliphatic. In addition, observed bands in the range of 1480 to 1720 cm^−1^ indicate the presence of CO_2_ groups related to ionized liquids in the structure. Absorption bands in the range of 680 and 980 cm^−1^ are assigned to Cr–O bond formation and aliphatic C–H groups, respectively. The presence of wide peaks shows the nature of Cr-MOF is nano-crystalline (confirmed by XRD). Based on the obtained results from the FTIR spectrum as well as the various configurations of 2,6-pyridine dicarboxylic acid ligand, the mechanism of Fig. 3 can be suggested for the formation of products. These reactions continue until it leads to the formation of the final structure of porous Cr-MOF. Figure 4 shows the proposed structure of the synthesized Cr-MOF. Using XRD and SEM data, the average size of Cr-MOF crystals was found to be about 26 nm. The porosity and pores of the structure volume were calculated using Barrett–Joyner–Halenda (BJH) technique. According to this method, the average pore diameter of the sample is in mesoporous nature (Fig. 5).

### Optimization of PT-µSPE

To investigate the parameters affecting PT-µSPE of parabens, different factors which potentially could affect the extraction were checked out employing two methods of one-variable-at-a-time and RSM. Aliquots of aqueous standard solution of each analyte at a concentration of 100.0 µg/L were used for optimization experiments. Each run was repeated three times.

#### Effect of type and volume of eluent solvent

After extraction of paraben by Cr-MOF, it is necessary to elute it using a proper solvent that can efficiently desorb the analytes. For selecting the best eluent, a number of common solvents including dichloromethane, carbon tetrachloride, 1,1,2,2-tetrachloroethane, dichloromethane, chloroform, methanol, 1-octanol, 1-hexanol, ethylbenzene, propyl benzene, benzyl alcohol and toluene were examined. Because of the highest analytical signal obtained, chloroform showed the best eluting performance and was selected as the eluent solvent for further experiments. In µPT-SPE technique, to achieve the highest efficiency, the volume of the eluting solvent should be kept in minimum; however, it should provide quantitative desorption of the analytes from the MOF surface. Volume of the eluent solvent, chloroform, was changed in the range of 50 to 400 μL. The results showed that the best response is achieved at 100 µL and after this point the absorbance decreased, probably because of the dilution effect when a greater volume of extraction solvent was used [28].

#### Number of elution cycles

The number of cycles of elution step is an important parameter that affects extraction efficiency [29]. The procedure of aspiration of a certain amount of the eluent solvent into the pipette tip and dispensed back into the same solvent tube is called an elution cycle. To investigate this parameter, 100 µL of chloroform was applied during cycles of 1 to 8. The best extraction efficiency was obtained by 4 cycles. Therefore, the number of elution cycles was selected 4 for the next experiments.

#### Response surface methodology

An optimization process was carried out to select the best experimental conditions for µSPE of parabens with RSM. RSM is a type of experimental design protocols to obtain an optimal extraction efficiency [30]. In this research, significant variables are assumed to be pH (A or X_1_), number of cycles of extraction (B or X_2_), volume of sample solution (C or X_3_) and amount of sorbent (mg) (D or X_4_). The p*K*a MP and PP are 8.2 and 8.4, respectively. To extract these compounds efficiently, the pH of the sample should be controlled to keep the selected compounds in their molecular state, that beneficial for extraction [5]. The low, middle and high levels of each factor were explained as − 1, 0, + 1, respectively. In Additional file 1: Table S1, the actual design of runs is explained.

In a system consist of four significant variables, A (X_1_), B (X_2_), C (X_3_) and D (X_4_), the mathematical relationship of the response on these variables can be approximated using quadratic (second degree) polynomial equation (Eq. 1):

1 $$ {\text{Y}} =\upbeta _{0} + \sum {\upbeta _{{\text{i}}} {\text{X}}_{{\text{i}}} } + \sum {\upbeta _{{{\text{ii}}}} {\text{X}}_{{{\text{ii}}}} } + \sum {\upbeta _{{{\text{ij}}}} {\text{X}}_{{\text{i}}} {\text{X}}_{{\text{j}}} } + {\text{e}}{.} $$

In Eq. 1, Y is predicted response, β_0_ is the constant, X_1_, X_2_, X_3_ and X_4_ are the coded independent variables, β_i_ is the linear effect, β_ii_ is the quadratic effect, β_ij_ proved the coefficient of the interaction factor, and e is the random error or allows for description or uncertainties between predicated and achieved data [31]. Equation 2 shows the mathematical relationship of the analytical signal and four indicated variables.

2 $$ \begin{aligned} {\text{R}} & = \left( { - 0.619} \right) + \left( {0.0400 \times {\text{A}}} \right) + \left( {0.0919 \times {\text{B}}} \right) + \left( {0.04318 \times {\text{C}}} \right) + \left( {0.1763 \times {\text{D}}} \right){-}\left( {0.01359{\text{A}}^{2} } \right) \\ & \quad - \left( {0.01139{\text{B}}^{2} } \right){-}\left( {0.000644{\text{C}}^{2} } \right){-}\left( {0.01777{\text{D}}^{2} } \right) + \left( {0.00485{\text{A}} \times {\text{B}}} \right){-}\left( {0.000233{\text{A}} \times {\text{C}}} \right) + \left( {0.00997{\text{A}} \times {\text{D}}} \right) \\ & \quad + \left( {0.000640{\text{B}} \times {\text{C}}} \right) + \left( {0.00017{\text{B}} \times {\text{D}}} \right) + \left( {0.000262{\text{C}} \times {\text{D}}} \right). \\ \end{aligned} $$

By solving these equation systems for the condition of ∂(Y)/∂(A) = 0, ∂(Y)/∂(B) = 0, ∂(Y)/∂(C) = 0 and ∂(Y)/∂(D) = 0, the critical point in the surface can be obtained [32]. In Additional file 1: Table S2, the summary of analysis of variance (ANOVA) is presented.

**Fig. 6:**
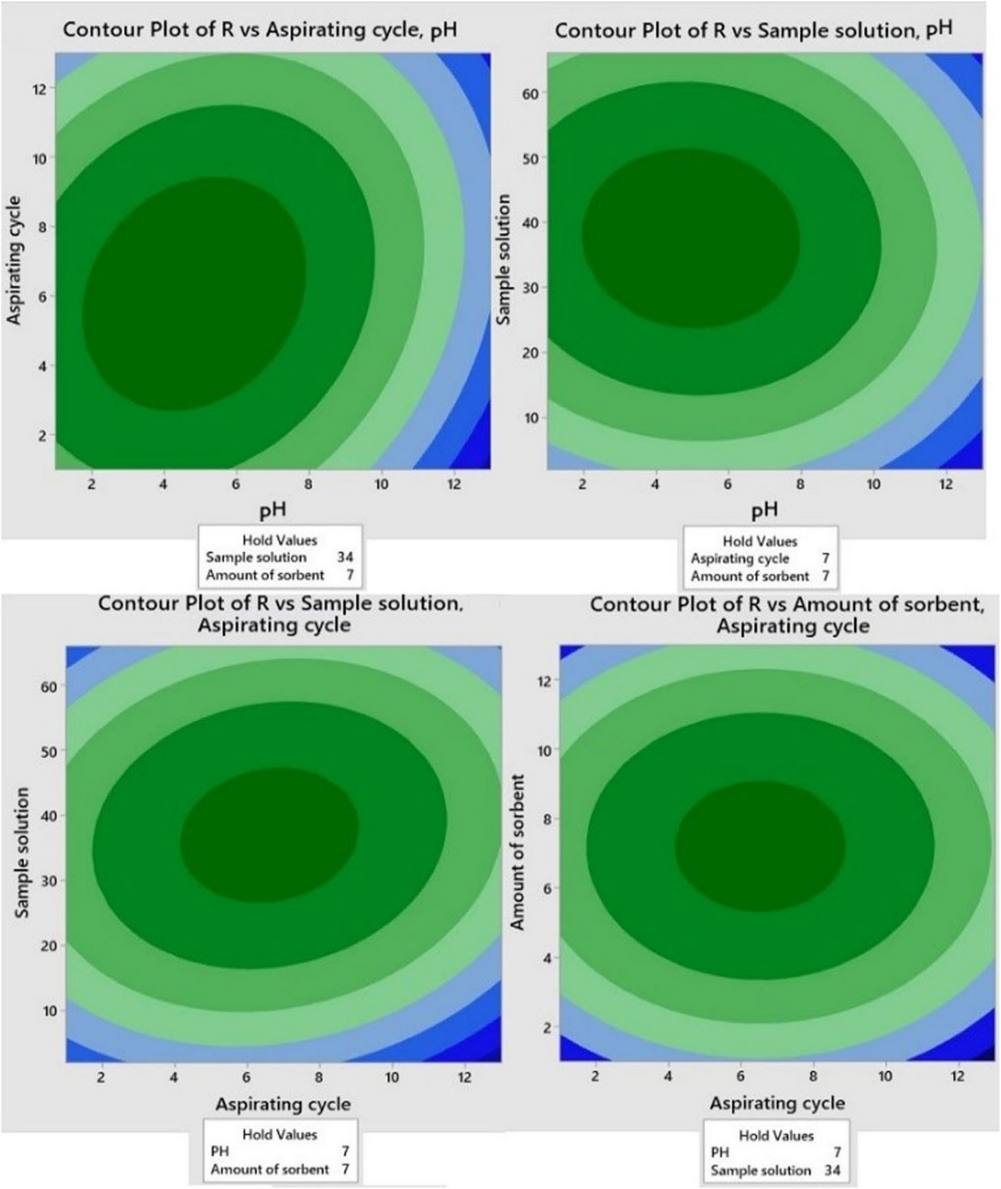
Response surface-2D/contours explaining the effect of independent variable on response (absorbance) of parabens

The achieved data for critical paint are as follows: pH (A) = 7, number of cycles of extraction (B) = 7, volume of sample solution (C) = 34 mL and amount of sorbent = 7 mg. The regression model for the analytes resulted in a determination coefficient (R^2^ = 0.9876), explaining that only 1.24% of the variation cannot be described with this model. The adjusted R^2^ = 0.9744 confirmed that this model was highly significant. Additionally, the prediction R^2^ of 0.9265 was an inacceptable agreement level of R^2^, thus the obtained prediction has a very good effectiveness. The F-value of model shows that the model is significant (Additional file 1: Table S2). A p-value lower than 0.0001 was found, demonstrating again the high significance of the regression models. A p-value less than 0.05 in the ANOVA table statistical significance of an effect at 95% confidence level. The F-value of Lack of fit (LOF) of 5.54 explained that the parameter was not significant relative to the pure errors. The small difference between the data predicted using the model and the experimental result, the analysis of variance of the model were highly satisfactory. The ANOVA of the regression model expressed that the quadratic model was significant, as was evident utilizing a very low probability value (p) (p_model_ ≤ 0.0001). Response surface-2D/contours explain the effect of independent variable on response of parabens (Fig. 6).

### Analytical performance

#### Linear range, limit of detection and enrichment factor

The calibration curves for parabens which were plotted separately indicated good linearity in the ranges of 1.0–200.0 µg/L for Parabens. The equation and regression coefficients were:

$$ {\text{A}} = 0.0099{\text{C}}\left( {{\upmu  g}/{\text{L}}} \right) + 0.0283\quad {\text{R}}^{2} = 0.9999\;{\text{for}}\;{\text{MP,}} $$

$$ {\text{A}} = 0.01{\text{C}}\left( {{\upmu  g}/{\text{L}}} \right) + 0.0064\quad {\text{R}}^{2} = 0.9998\;{\text{for}}\;{\text{PP,}} $$

where C and A are the concentration of parabens and analytical signal, respectively.

The percent extraction (E%) of analytes was obtained from the equation E% = 100(C_B_/C_A_). C_A_ and C_B_ are concentration the analyte in solution before and after extraction, respectively.

The detection limits (LOD) and limit of quantitation (LOQ) of the analytes were calculated based on 3S_b_/m and 10S_b_/m (In the equation, S_b_ is standard deviation of 10 consecutive measurements of the blank extracted with the same procedure and m is the slope of calibration curve). LODs of 0.25 µg/L and 0.24 µg/L were obtained for MP and PP respectively. LOQs were 0.83 (for MP) and 0.80 (for PP). Considering the final elution volume of 100 μL and the sample volume of 34 mL, an enrichment factor (EF) of 340 folds was expected to be achieved, which is close to the EF experimentally observed (330 fold) with an extraction efficiency of 97%. Reproducibility of the protocol as RSD% (n = 5) for concentration of 20 µg/L of the analytes was 2.95% (for MP) and 5.78% (for PP). Adsorptive capacity of 200 µg of each paraben per 6.0 mg of Cr-MOF was achieved for the uptake of both parabens after reaching saturation. Table 1 compares the characteristic data of the present method to those recently reported in the literature for the same analytes.

**Table 1:** Comparison of the published protocol for MP and PP parabens determination with proposed technique in the research

Sample	Analyte(s)	Extraction method	Instrument	LOD (µg/L)	LOQ (µg/L)	Linear range (µg/L)	Refs.
Water, cosmetic creams, human urine	Methyl, ethyl, propyl, isopropyl, butyl and isobutyl and benzyl paraben	SPME	HPLC- UV	1.5–2.6	5.0–8.7	0.5–147.0	[1]
Human urine	Methyl, ethyl, propyl and butyl paraben	SPME	HPLC- UV	0.03–0.04 (µg/g)	0.40–0.97 (µg/g)	0.10–10.00 (µg/g)	[5]
River water, mouthwash, hand cream	Methyl, ethyl, propyl, isopropyl and butyl paraben	SPME	GC	0.0002–0.0500	Not mentioned	50.0–300.0	[6]
Wastewater and shampoo	Methyl and propyl paraben	PT-µSPE	Spectrophotometer	0.24–0.25	0.80–0.83	1.0–200.0	This work

*SPME* solid phase microextraction, *HPLC–UV* high performance liquid chromatography–ultraviolet detection, *GC* gas chromatography

#### Repeated usage

The synthesized Cr-MOF could be reused at least 5 times for the parabens extraction in various samples. It was also kept in dried air for at least 8 months without losing its extraction efficiency. The repeatability between different batches of Cr-MOF (n = 5) was achieved to be better than 5.6%.

#### Selectivity of Cr-MOF toward parabens

**Fig. 7:**
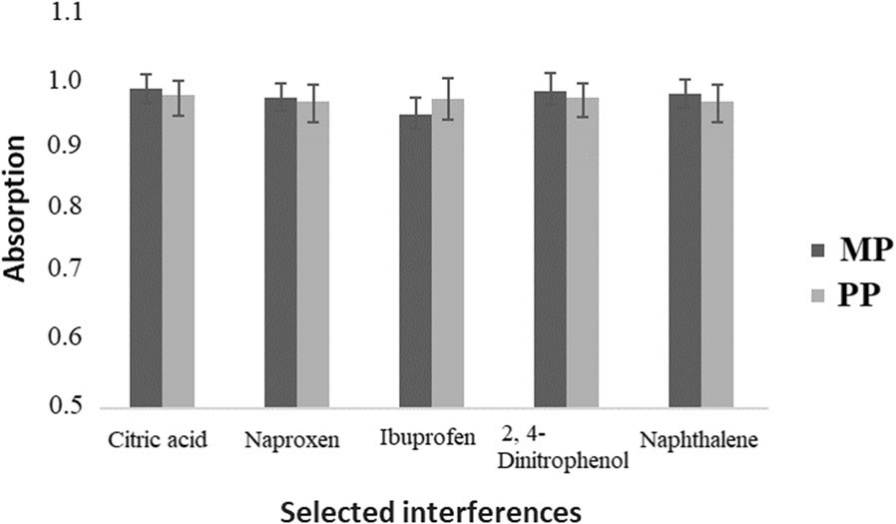
Competitive bar graph of absorbance of MP and PP (100 µg/L) in the presence of other potential interfering compounds with a concentration of 10 mg/L

To study the selectivity Cr-MOF for the extraction of MP and PP in samples including similar analytes, aliquots of 34 mL of aqueous sample consist of 100 µg/L of each of the analytes and potential interferences was taken and the selected protocol was performed. Naphthalene, 2,4-dinitrophenol, ibuprofen and naproxen that are normally present in natural sources were chosen for this purpose. No interferences were observed (Fig. 7).

#### Real samples analysis

**Fig. 8:**
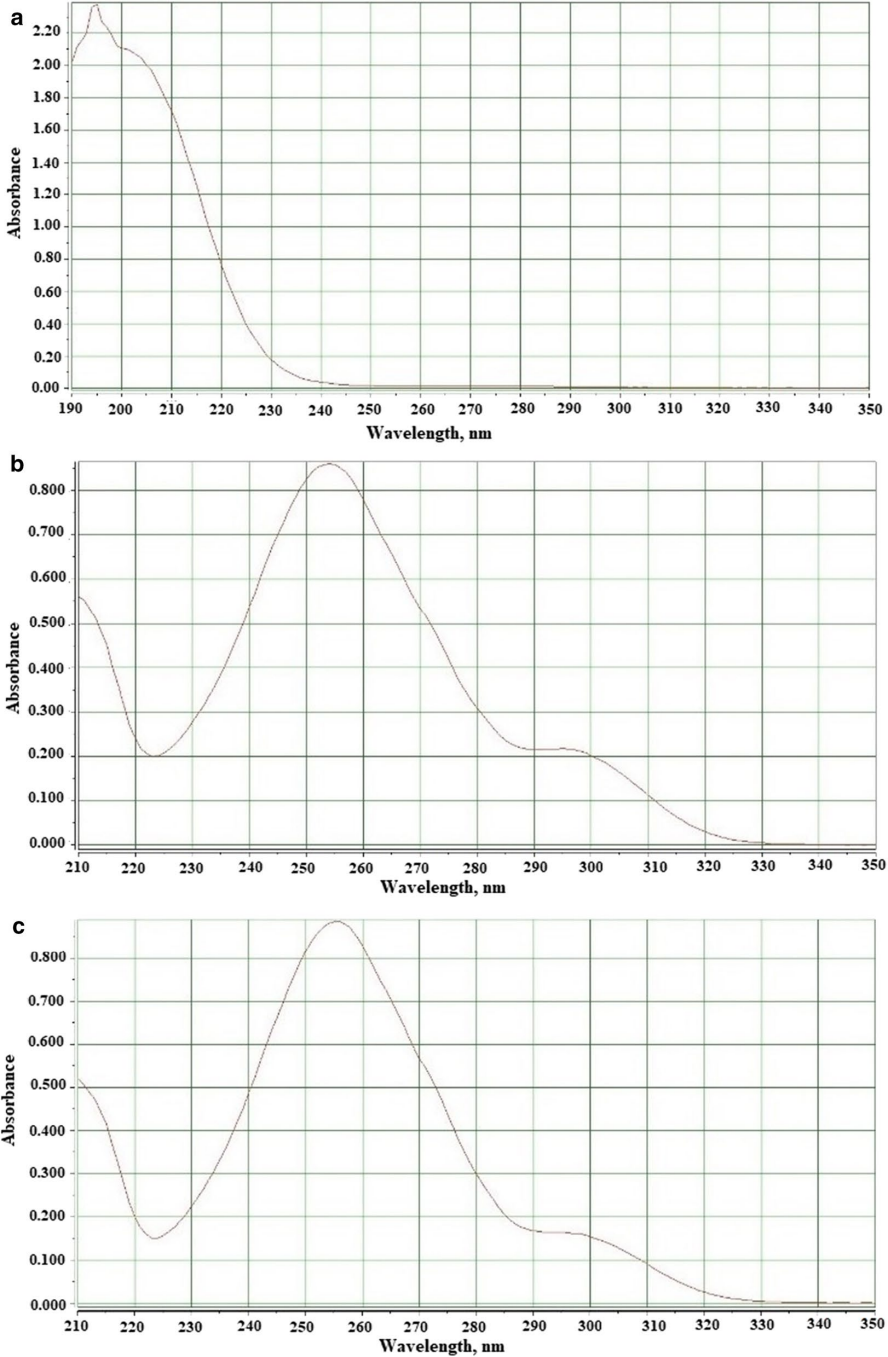
Absorption spectra of extracted methyl and propyl parabens from wastewater before (**a**) and after spiking with 85 µg/L of the analytes (**b** methyl paraben, **c** propyl paraben)

To investigate the effect of sample media on analytical signal, both samples were separately spiked at three concentration levels of 20, 85 and 140 µg/L with the selected analytes. Recoveries of parabens from different real samples applying the present technique are showed in Table 2. Recoveries of the analytes were between 95.7 and 102.3% (for MP) and 95.4–101.9% (for PP) that indicate negligible effect of the sample matrix on the determination of these analyte with suggested technique. These observations excesses that the suggested procedure can be employed to the analysis of MP and PP in complicated matrices. Figure 8 shows the absorption spectra of extracted wastewater before and after spiking with 85 µg/L of the analytes.

**Table 2:** Recovery and reproducibility results for determination of parabens achieved for two real samples

Analyte added	Sample	Analyte added (µg/L)	Analyte found (µg/L)	Recovery	RSD% (n = 3)
MP	Wastewater	–	–	–	–
		20	19.3	96.5	1.6
		85	86.0	101.1	1.4
		140	134.0	95.7	3.6
MP	Shampoo	–	–	–	
		20	20.0	100.0	4.1
		85	87.0	102.3	3.4
		140	134.6	96.1	2.2
PP	Wastewater	–	–	–	–
		20	20.0	100.0	2.1
		85	81.6	96.0	2.4
		140	135.0	96.4	1.9
PP	Shampoo	–	–	–	–
		20	20.0	100.0	1.1
		85	86.6	101.9	1.3
		140	133.5	95.4	2.3

## Conclusion

A new sorbent, Cr-MOF, with a huge surface area was synthesized, characterized and applied for pipette tip micro-solid phase extraction of methyl- and propyl-parabens in wastewater and shampoo samples with high recovery and low detection limit. No special sample pre-treatment was required and pipette tip column was simply packed with the sorbent and no additional steps were required before passing sample through it. The linear range covers wide concentrations, and Cr-MOF could selectively extract parabens for analysis even at the trace concentrations. The adsorbent could be used for at least 20 extractions without substantial change in its adsorption power. The PT-SPE column needs only 6 mg of the sorbent and the total analysis time was less than 6 min. Due to the application of a conventional spectrophotometer and no sample pre-treatment or additional reagents, this method is very simple and extremely economical with easy applicability for routine analysis.

## Supplementary Information

**Additional file 1: Table S1.** Design media in the RSM model for the optimization of Cr-MOF PT-µSPE. **Table S2.** Analysis of variance (ANOVA) for parabens.

## Data Availability

The majority of the data used to support the findings of this study are included within the article. Other data are available from the corresponding author upon request.

## Abbreviations

MP: Methylparaben
PP: Propylparaben
HPLC–UV: High performance liquid chromatography–ultraviolet
DAD: Diode array detection
UV: Ultraviolet
GC: Gas chromatography
GC–MS: Gas chromatography–mass spectrometry
SPE: Solid phase extraction
MOFs: Metal–organic frameworks
RSM: Response surface methodology
MOF PT-µSPE: Metal organic frameworks pipette tip micro-solid phase extraction
XRD: X-ray diffraction
SEM: Scanning electron microscope
ANOVA: Analysis of variance
LOD: Detection limits
EF: Enrichment factor
SPME: Solid phase microextraction
HPLC: High performance liquid chromatography
