# Correction to: Chromium‑based metal organic framework for pipette tip micro‑solid phase extraction: an effective approach for determination of methyl and propyl parabens in wastewater and shampoo samples

**DOI:** 10.1186/s13065-021-00790-x

**Published:** 2021-12-13

**Authors:** Massoud Kaykhaii, Sayyed Hossein Hashemi, Fariba Andarz, Amin Piri, Ghasem Sargazi

**Affiliations:** 1 Department of Chemistry, Faculty of Sciences, University of Sistan and Baluchestan, 98136-674 Zahedan, Iran; 2 Department of Marine Chemistry, Faculty of Marine Science, Chabahar Maritime University, Chabahar, Iran; 3 Nanomaterial Technology Department, Non-Communicable Diseases Research Centre, Bam University of Medical Sciences, Bam, Iran; 4 Department of Process Engineering and Chemical Technology, Faculty of Chemistry, Gdansk University of Technology, Gdansk, Poland

## Correction to: BMC Chem (2021) 15:60 https://doi.org/10.1186/s13065-021-00786-7

Following publication of the original article [1], the author group was incorrectly published with the inclusion of the last author “Grzegorz Boczkaj” who did not approve to be author of the article. Hence, the article was corrected with the following author groups and updated author contribution statement:

Author list: Massoud Kaykhaii, Sayyed Hossein Hashemi, Fariba Andarz, Amin Piri and Ghasem Sargazi.

Authors’ contributions: MK planned the study and wrote the manuscript. SHH co-wrote and updated the manuscript. FA did the practical work. AP did the chemometrics, GS synthesized MOF. All authors read and approved the final manuscript.

The original article has been corrected.
