# Supplementary material for: Chromium-based metal organic framework for pipette tip micro-solid phase extraction: an effective approach for determination of methyl and propyl parabens in wastewater and shampoo samples
Source: BMC Chem. 2021 Nov 6;15(1):60. doi: 10.1186/s13065-021-00786-7 (PMC8571821; doi:10.1186/s13065-021-00786-7)
Supplement: Supplementary file 1 — Additional file 1: Table S1. Design media in the RSM model for the optimization of Cr-MOF PT-µSPE. Table S2. Analysis of variance (ANOVA) for parabens. [file 13065_2021_786_MOESM1_ESM.docx]

**Additional file 1 to:**

**Chromium-based metal organic framework for pipette tip micro-solid phase extraction – an effective approach for determination of methyl and propyl parabens in wastewater and shampoo samples**

Massoud Kaykhaii^1,4^[[1]](#footnote-1)^^, Sayyed Hossein Hashemi^2^, Fariba Andarz^1^, Amin Piri^1^, Ghasem Sargazi^3^ and Grzegorz Boczkaj^4^

**Table S1**. Design media in the RSM model for the optimization of Cr- MOF PT-µSPE

| **No.** | **A** | **B** | **C** | **D** | **StdOrder** | **RunOrder** | **PtType** | **Blocks** | **R** | **FITS1** | **RESI1** | **SRES1** | **TRES1** | **HI1** | **COOK1** | **DFIT1** |
| --- | --- | --- | --- | --- | --- | --- | --- | --- | --- | --- | --- | --- | --- | --- | --- | --- |
| 1 | 7 | 7 | 34 | 7 | 20 | 1 | 0 | 1 | 1.08823 | 1.09698 | -0.0087498 | -0.20357 | -0.19646 | 0.183333 | 0.000581 | -0.09308 |
| 2 | 10 | 10 | 18 | 4 | 4 | 2 | 1 | 1 | 0.24276 | 0.20950 | 0.0332622 | 1.10578 | 1.11538 | 0.600000 | 0.114633 | 1.36606 |
| 3 | 4 | 10 | 50 | 4 | 7 | 3 | 1 | 1 | 0.87744 | 0.79556 | 0.0818785 | 2.72200 | 3.82292 | 0.600000 | 0.694622 | 4.68210 |
| 4 | 4 | 10 | 50 | 10 | 15 | 4 | 1 | 1 | 0.67999 | 0.68912 | -0.0091235 | -0.30331 | -0.29324 | 0.600000 | 0.008624 | -0.35914 |
| 5 | 8 | 5 | 1 | 1 | 8 | 5 | 1 | 1 | 0.34341 | 0.35394 | -0.0105300 | -0.35006 | -0.33882 | 0.600000 | 0.011488 | -0.41496 |
| 6 | 1 | 6 | 1 | 1 | 1 | 6 | 1 | 1 | 0.82215 | 0.82241 | -0.0002528 | -0.00840 | -0.00810 | 0.600000 | 0.000007 | -0.00992 |
| 7 | 10 | 7 | 1 | 1 | 10 | 7 | 1 | 1 | 0.41030 | 0.44689 | -0.0365961 | -1.21662 | -1.23973 | 0.600000 | 0.138765 | -1.51835 |
| 8 | 3 | 8 | 1 | 1 | 3 | 8 | 1 | 1 | 0.59941 | 0.60643 | -0.0070157 | -0.23323 | -0.22519 | 0.600000 | 0.005100 | -0.27580 |
| 9 | 11 | 9 | 1 | 1 | 11 | 9 | 1 | 1 | 0.48063 | 0.44962 | 0.0310075 | 1.03083 | 1.03332 | 0.600000 | 0.099619 | 1.26555 |
| 10 | 12 | 11 | 1 | 1 | 12 | 10 | 1 | 1 | 0.39681 | 0.41155 | -0.0147427 | -0.49011 | -0.47639 | 0.600000 | 0.022520 | -0.58345 |
| 11 | 9 | 11 | 1 | 1 | 9 | 11 | 1 | 1 | 0.64403 | 0.65943 | -0.0153960 | -0.51183 | -0.49789 | 0.600000 | 0.024560 | -0.60979 |
| 12 | 16 | 12 | 1 | 1 | 16 | 12 | 1 | 1 | 0.65189 | 0.60635 | 0.0455351 | 1.51379 | 1.59510 | 0.600000 | 0.214834 | 1.95359 |
| 13 | 19 | 13 | 0 | 1 | 19 | 13 | 0 | 1 | 1.08823 | 1.09698 | -0.0087498 | -0.20357 | -0.19646 | 0.183333 | 0.000581 | -0.09308 |
| 14 | 14 | 14 | 1 | 1 | 14 | 14 | 1 | 1 | 0.49986 | 0.51878 | -0.0189103 | -0.62866 | -0.61453 | 0.600000 | 0.037051 | -0.75264 |
| 15 | 17 | 15 | 0 | 1 | 17 | 15 | 0 | 1 | 1.04757 | 1.09698 | -0.0494089 | -1.14956 | -1.16404 | 0.183333 | 0.018541 | -0.55153 |
| 16 | 6 | 16 | 1 | 1 | 6 | 16 | 1 | 1 | 0.28681 | 0.27254 | 0.0142749 | 0.47456 | 0.46102 | 0.600000 | 0.021113 | 0.56463 |
| 17 | 2 | 17 | 1 | 1 | 2 | 17 | 1 | 1 | 0.23421 | 0.25101 | -0.0168025 | -0.55859 | -0.54437 | 0.600000 | 0.029252 | -0.66672 |
| 18 | 13 | 18 | 1 | 1 | 13 | 18 | 1 | 1 | 0.78802 | 0.77600 | 0.0120202 | 0.39960 | 0.38728 | 0.600000 | 0.014970 | 0.47432 |
| 19 | 18 | 19 | 0 | 1 | 18 | 19 | 0 | 1 | 1.08646 | 1.09698 | -0.0105170 | -0.24469 | -0.23630 | 0.183333 | 0.000840 | -0.11196 |
| 20 | 5 | 20 | 1 | 1 | 5 | 20 | 1 | 1 | 0.87744 | 0.88862 | -0.0111833 | -0.37178 | -0.36004 | 0.600000 | 0.012958 | -0.44096 |
| 21 | 23 | 21 | -1 | 2 | 23 | 21 | -1 | 2 | 0.72417 | 0.67807 | 0.0461011 | 1.63843 | 1.75614 | 0.650000 | 0.311586 | 2.39322 |
| 22 | 29 | 22 | 0 | 2 | 29 | 22 | 0 | 2 | 1.08411 | 1.02387 | 0.0602427 | 1.44660 | 1.51152 | 0.233333 | 0.039806 | 0.83387 |
| 23 | 27 | 23 | -1 | 2 | 27 | 23 | -1 | 2 | 0.30729 | 0.33943 | -0.0321375 | -1.14216 | -1.15578 | 0.650000 | 0.151418 | -1.57506 |
| 24 | 26 | 24 | -1 | 2 | 26 | 24 | -1 | 2 | 0.45259 | 0.49489 | -0.0423026 | -1.50343 | -1.58207 | 0.650000 | 0.262355 | -2.15600 |
| 25 | 28 | 25 | -1 | 2 | 28 | 25 | -1 | 2 | 0.44164 | 0.42886 | 0.0127811 | 0.45424 | 0.44098 | 0.650000 | 0.023949 | 0.60095 |
| 26 | 21 | 26 | -1 | 2 | 21 | 26 | -1 | 2 | 0.83038 | 0.86167 | -0.0312892 | -1.11201 | -1.12227 | 0.650000 | 0.143531 | -1.52940 |
| 27 | 25 | 27 | -1 | 2 | 25 | 27 | -1 | 2 | 0.25682 | 0.23387 | 0.0229463 | 0.81551 | 0.80520 | 0.650000 | 0.077193 | 1.09730 |
| 28 | 30 | 28 | 0 | 2 | 30 | 28 | 0 | 2 | 1.04105 | 1.02387 | 0.0171828 | 0.41261 | 0.40004 | 0.233333 | 0.003238 | 0.22069 |
| 29 | 22 | 29 | -1 | 2 | 22 | 29 | -1 | 2 | 0.21945 | 0.20752 | 0.0119329 | 0.42409 | 0.41131 | 0.650000 | 0.020876 | 0.56053 |
| 30 | 24 | 30 | -1 | 2 | 24 | 30 | -1 | 2 | 0.48421 | 0.54967 | -0.0654575 | -2.32635 | -2.86218 | 0.650000 | 0.628166 | -3.90050 |

**Table S2**. Analysis of variance (ANOVA) for parabens

| **Source** | **DF** | **Adj SS** | **Adj MS** | **F-Value** | **P-Value** |
| --- | --- | --- | --- | --- | --- |
| Model | 15 | 2.53198 | 0.168799 | 74.62 | 0.000 |
| Blocks | 1 | 0.03564 | 0.035635 | 15.75 | 0.001 |
| Linear | 4 | 0.78080 | 0.195201 | 86.29 | 0.000 |
| pH (A) | 1 | 0.64188 | 0.641879 | 283.76 | 0.000 |
| Aspirating cycle (B) | 1 | 0.02473 | 0.024730 | 10.93 | 0.005 |
| Sample solution (C) | 1 | 0.10220 | 0.102196 | 45.18 | 0.000 |
| Amount of sorbent (D) | 1 | 0.01200 | 0.011998 | 5.30 | 0.037 |
| Square | 4 | 1.53664 | 0.384161 | 169.83 | 0.000 |
| pH × pH | 1 | 0.41038 | 0.410380 | 181.42 | 0.000 |
| Aspirating cycle × Aspirating cycle | 1 | 0.28817 | 0.288169 | 127.39 | 0.000 |
| Sample solution × Sample solution | 1 | 0.74557 | 0.745574 | 329.60 | 0.000 |
| Amount of sorbent × Amount of sorbent | 1 | 0.70157 | 0.701569 | 310.15 | 0.000 |
| 2-Way Interaction | 6 | 0.17890 | 0.029817 | 13.18 | 0.000 |
| pH × Aspirating cycle | 1 | 0.03044 | 0.030439 | 13.46 | 0.003 |
| pH × Sample solution | 1 | 0.00200 | 0.001998 | 0.88 | 0.363 |
| pH × Amount of sorbent | 1 | 0.12878 | 0.128781 | 56.93 | 0.000 |
| Aspirating cycle × Sample solution | 1 | 0.01511 | 0.015109 | 6.68 | 0.022 |
| Aspirating cycle × Amount of sorbent | 1 | 0.00004 | 0.000038 | 0.02 | 0.899 |
| Sample solution × Amount of sorbent | 1 | 0.00254 | 0.002536 | 1.12 | 0.308 |
| Error | 14 | 0.03167 | 0.002262 |  |  |
| Lack-of-Fit | 10 | 0.02954 | 0.002954 | 5.54 | 0.057 |
| Pure Error | 4 | 0.00213 | 0.000533 |  |  |
| Total | 29 | 2.56365 |  |  |  |

1. Corresponding author. Tel: +98 (54) 33446413; Fax: +98 (54) 33431067; E-mail: kaykhaii@chem.usb.ac.ir

   ^1^ Department of Chemistry, Faculty of Sciences, University of Sistan and Baluchestan, Zahedan 98136-674, Iran

   ^2^Department of Marine Chemistry, Faculty of Marine Science, Chabahar Maritime University, Chabahar, Iran

   ^3^Nanomaterial Technology Department, Non-communicable Diseases Research Centre, Bam University of Medical Sciences, Bam, Iran

   ^4^Department of Process Engineering and Chemical Technology, Faculty of Chemistry, Gdansk University of Technology, Gdansk, Poland [↑](#footnote-ref-1)
